# Domestic Work and Psychological Distress−What Is the Importance of Relative Socioeconomic Position and Gender Inequality in the Couple Relationship?

**DOI:** 10.1371/journal.pone.0038484

**Published:** 2012-06-13

**Authors:** Lisa Harryson, Mattias Strandh, Anne Hammarström

**Affiliations:** 1 Department of Public Health and Clinical Medicine, Family Medicine, Umeå University, Umeå, Sweden; 2 National Graduate School for Gender Studies at Umeå Centre for Gender Studies, Umeå University, Umeå, Sweden; 3 Department of Sociology, Umeå University, Umeå, Sweden; 4 Umeå Centre for Gender Studies in Medicine, Umeå University, Umeå, Sweden; Federal University of Rio de Janeiro, Brazil

## Abstract

**Aims:**

The aim of this study was to investigate whether the relation between responsibility for domestic work and psychological distress was influenced by perception of gender inequality in the couple relationship and relative socioeconomic position.

**Methods:**

In the Northern Swedish Cohort, all pupils who studied in the last year of compulsory school in a northern Swedish town in 1981 have been followed regularly until 2007. In this study, participants living with children were selected (n = 371 women, 352 men). The importance of relative socioeconomic position and perception of gender inequality in the couple relationship in combination with domestic work for psychological distress was examined through logistic regression analysis.

**Results:**

Two combinations of variables including socioeconomic position (‘having less than half of the responsibility for domestic work and partner higher socioeconomic position’ and ‘having more than half the responsibility for domestic work and equal socioeconomic position’) were related to psychological distress. There were also higher ORs for psychological distress for the combinations of having ‘less than half of the responsibility for domestic work and gender-unequal couple relationship’ and ‘more than half the responsibility for domestic work and gender-unequal couple relationship’. Having a lower socioeconomic position than the partner was associated with higher ORs for psychological distress among men.

**Conclusions:**

This study showed that domestic work is a highly gendered activity as women tend to have a greater and men a smaller responsibility. Both these directions of inequality in domestic work, in combination with experiencing the couple relationship as gender-unequal, were associated with psychological distress There is a need for more research with a relational approach on inequalities in health in order to capture the power relations within couples in various settings.

## Introduction

In earlier health research on domestic work, studies have mainly focused on women, whereas the importance of socioeconomic position has only been studied to a limited extent [1]–[5]. It is well documented that women perform most of the unpaid domestic work, irrespective of their amount of paid work [6], [7] and that gender inequality in domestic work contributes to gender differences in psychological distress, with women having higher levels of distress than men[8]–[10]. One of the few studies taking gender and socioeconomic position into account showed that women with a high level of responsibility for domestic work and a low socioeconomic position had higher risks of psychological distress [5], indicating that a lower socioeconomic position can be related to higher involvement in domestic work among women [7]. However, new ways of measuring socioeconomic positions that can be of importance for domestic work have been suggested; measurements that extend the traditional individual measure of socioeconomic position to consider dimensions of power and gender relations as well [11], [12]. It is indicated that the relative socioeconomic status between the partners can influence the division of domestic work; the division tends to be more equal when the couple’s socioeconomic position is equal or when the women have a higher socioeconomic position than the partner [13]. When women’s and men’s lives are more similar in terms of higher socioeconomic position, some of the gender inequality in domestic work may be neutralized or levelled out [7]. Therefore the relative socioeconomic position may have a potential modifying effect on relations between domestic work and health status [2].

Overall, there are few theoretical explanations for how domestic work and couple relationships are related to health [14]. It has been suggested that the experience of unfairness could be a moderating mechanism between inequality in domestic work and psychological distress. Experiencing the division of domestic work as not only unequal, but also unfair, could increase the risk of psychological distress [15]. One approach to capture the experience of unfairness in the domestic sphere is to investigate the perception of gender inequality in the couple relationship [16], which in one of our earlier studies has been shown to be associated with psychological distress [10]. Another theoretical explanation for how domestic work and couple relationships are related to health is the social relations of gender, which implies that the division of domestic work and the experience of gender equality are related to normative expectations of how women and men should act [17], [18]. However, we could find no studies of domestic work and psychological distress that include perception of gender equality in the couple relationship or relative socioeconomic position in couples.

The aim of this study was to investigate whether the relation between responsibility for domestic work and psychological distress was influenced by perception of gender inequality in the couple relationship and relative socioeconomic position.

## Methods

### Ethics Statement

The Regional Ethical Review Board in Umeå, Sweden, has approved this study.

### Sample and Data Collection

The population of this study is the Northern Swedish Cohort, which consist of all pupils (n = 1083; 506 girls and 577 boys) who studied or should have studied in their last year of compulsory school in a medium-sized Swedish industrial town in 1981. The participants were invited to answer a comprehensive questionnaire concerning school, employment, socioeconomic conditions, health and health behaviour. The first and second rounds of data collection in 1981 and in 1983 were carried out at the pupils’ school. The participants have subsequently been invited to their former compulsory school in order to answer a similar questionnaire in 1986, 1995 and 2007. Those who could not attend these occasions were sent a questionnaire by mail and a reminder if they did not respond [19].

In this study, data from when participants were 21 (1986) and 42 years old (2007) have been analysed. The participation rate (in relation to those still alive in the original cohort) was 98% in 1986 and 94% in 2007. There were 21 couples in the cohort. In order to attain independency between the cases, one partner of each couple (n = 21) was randomly excluded. As the presence of children increases the amount of domestic work for both women and men [13], a selection of participants was made (based on questionnaire data at age 42) who at age 42 lived together with children. This resulted in a total sample of 723 participants (371 women and 352 men). All other participants (n = 267) were excluded from the analysis.

### Measures

#### Exposure variables

“Perceptions of gender equality in the couple relationship” were measured with the question “How gender-equal do you consider your couple relationship to be?” [16] “Gender-equal” was used as reference category compared to “not gender-equal”.

“Responsibility for domestic work” (age 42) was measured with the question “How much of the responsibility for the domestic work do you take?” The answer alternative “half” was used as reference category compared to “none”, “less than half”, “more than half” and “all”. In order to reduce the number of categories in the combination variables and to increase the statistical power in the analysis, responsibility for domestic work was categorized into three groups: half, less than half (i.e. those who answered none or less than half) and more than half (i.e. i.e. those who answered more than half or all).

A new variable was then created by combining the two variables “responsibility for domestic work” and “perceptions of gender equality in the couple relationship” into six categories (0 =  half and gender-equal; 1 =  half and not gender-equal; 2 =  less than half and gender-equal; 3 =  less than half and not gender-equal; 4 = more than half and gender-equal; 5 =  more than half and not gender-equal), with 0 as the reference category.

“Relative socioeconomic position” (age 42) was created by “individual socioeconomic position” (age 42) and “partner socioeconomic position” (age 42), measured with occupation level on the basis of the Swedish SEI classification [20]. Upper white-collar workers (including self-employed 46 to 99) was used as reference category compared to lower white-collar (33 to 45) and blue-collar workers (11 to 32). Equal socioeconomic position to the partner was used as reference category (0) compared to higher socioeconomic position than the partner (1) and lower socioeconomic position than the partner (2).

A new variable was created by combining the two variables, “responsibility for domestic work” and “relative socioeconomic position” (0 =  half and equal socioeconomic position; 1 =  half and individual higher socioeconomic position; 2 =  half and partner higher socioeconomic position; 3 =  less than half and equal socioeconomic position; 4 =  less than half and individual higher socioeconomic position; 5 =  less than half and partner higher socioeconomic position; 6 =  more than half and equal socioeconomic position; 7 =  more than half and individual higher socioeconomic position; 8 =  more than half and partner higher socioeconomic position), with 0 as reference category.

#### Background variables

“Gender” was measured as women = 0, men = 1.

“Number of children” was measured with a question regarding the number of children that the participants lived with (1 to ≥4 children).

#### Outcome variable

“Psychological distress” at age 42 was chosen as the outcome variable and measured by an index consisting of six items (restlessness, concentration problems, worries/nervousness, palpitations, anxiety and other nervous distress) that the participants had felt during the last 12 months (range 0–6 with higher values corresponding to more psychological distress). The index was not normally distributed and therefore dichotomized. A cut-off point at the 75th percentile (0 =  no distress, 1 =  one or more items of distress) was chosen in accordance with earlier research [21] and in order to achieve statistical power. The questions were derived from the Swedish Survey of Living Conditions [22].

#### Indicator of health-related selection

“Psychological distress at age 21″ was used as an indicator of health-related selection, measured with the same questions and dichotomized in the same way as at age 42.

### Statistical Analysis

All statistical analyses were performed using PASW Statistics 18 with a significance level at 0.05. Prevalence proportions (percentages) of the different characteristic of the study population were calculated, and differences between women and men were analysed using χ^2^ test (Table 1). Crude and multivariate stepwise logistic regression analysis for psychological distress (age 42) was performed in relation to responsibility for domestic work, relative socioeconomic position and perception of gender equality in the couple relationship (Table 2). The analysis was performed separately for women and men. The combined variables “responsibility for domestic work and perception of gender equality in the couple relationship” (Table 3) and “responsibility for domestic work and relative socioeconomic position” (Table 4) were analysed with stepwise logistic regression analysis. Due to low statistical power, it was not possible to perform separate analyses for women and men in the analysis of the combined variables. However, adjustments were made for gender and also for psychological distress at age 21. The results of the logistic regression analysis are presented as odds ratios (ORs) with 95% confidence intervals (CIs).

**Table 1:** Distribution in percentage of psychological distress (age 21 and 42), responsibility for domestic work, perception of gender equality in the couple relationship, individual, partner and relative socioeconomic position.

	Women	Men	p-value*		Total
Variables	n = 371	n = 352		χ^2^ value	n = 723
**Psychological distress (age 21)**	32.2	26.1	0.07	3.2	29.2
**Psychological distress (age 42)**	38.8	26.5	<0.001	14.4	32.8
**Responsibility for domestic work**					
None	0.0	0.3	<0.001	240.0	0.1
Less than half	2.7	34.2			18.0
Half	27.0	46.7			36.3
More than half	56.3	9.7			33.7
All	14.0	9.1			11.6
**Perception of gender equality in the couple relationship**					
Gender-equal	34.4	44.3	0.01	6.8	39.2
Not gender-equal	65.6	55.7			60.8
**Individual socioeconomic position**					
Upper white-collar	51.5	56.7	0.002	12.2	54.0
Lower white-collar	18.1	9.1			13.7
Blue-collar	30.5	34.2			32.3
**Partner socioeconomic position**					
Upper white-collar	55.7	52.8	0.20	3.23	54.3
Lower white-collar	8.5	12.7			10.5
Blue-collar	35.9	34.5			35.2
**Relative socioeconomic position**					
Equal to partner	54.2	59.8	0.24	2.98	56.9
Higher than partner	23.3	18.4			20.9
Lower than partner	22.4	21.8			22.1

* Significance differences tested by χ^2^.

## Results

Statistically significantly more women than men reported psychological distress at age 42, but no gender differences were found at age 21 (Table 1). Women had a higher level of responsibility for domestic work than men, and more women than men were lower white-collar workers. More men than women perceived their couple relationship as gender-equal. Partner’s socioeconomic position and relative socioeconomic position was equally distributed among women and men, with more than half of the population having an equal socioeconomic position to their partner.

In the crude logistic regression analysis (Table 2, Model 1), having the whole responsibility for domestic work was associated with higher ORs for psychological distress among women, and having less than half of the responsibility for domestic work was associated with psychological distress for men. There were no statistically significant associations between relative socioeconomic position and psychological distress among women, while a lower socioeconomic position than the partner was associated with psychological distress for men in the crude logistic regression analysis. Perceiving the couple relationship as not gender-equal was associated with psychological distress for both women and men. Model 2 in Table 2 shows the multivariate regressions. There were no associations between responsibility for domestic work and psychological distress for women, as relative socioeconomic position (rather than psychological distress at age 21) made the association statistically insignificant. For men, having less than half of the responsibility for domestic work and a lower socioeconomic position than the partner was associated with psychological distress.

**Table 2:** Logistic regression analysis for psychological distress (age 42) for three different sets of independent variables (ORs and 95% CIs).

	Model 1		Model 2	
	OR	95% CI	OR	95% CI
**WOMEN**				
**Responsibility for domestic work**				
Half (reference)	1.00	-	1.00	-
None†	-	-	-	-
Less than half†	-	-	-	-
More than half	1.09	0.66−1.80	0.98	0.59−1.66
All	2.65*	1.33−5.27	1.68	0.71−3.97
**Relative socioeconomic position**				
Equal to partner (reference)	1.00		1.00	
Higher than partner	0.76	0.44−1.33	0.73	0.41−1.30
Lower than partner	0.76	0.43−1.33	0.77	0.43−1.38
**Perception of gender equality in the couple relationship**				
Gender-equal (reference)	1.00			
Not gender-equal	1.86*	1.14−3.02		
**MEN**				
**Responsibility for domestic work**				
Half (reference)	1.00		1.00	
None†	-	-	-	-
Less than half	2.14*	1.26−3.65	2.30*	1.31−4.00
More than half	0.66	0.24−1.83	0.67	0.23−1.95
All	2.10	0.92−4.80	1.76	0.29−10.48
**Relative socioeconomic position**				
Equal to partner (reference)	1.00	-	1.00	-
Higher than partner	0.75	0.36−1.56	0.74	0.35−1.58
Lower than partner	1.93*	1.07−3.48	1.92*	1.04−3.54
**Perception of gender equality in the couple relationship**				
Gender-equal (reference)	1.00			
Not gender-equal	1.86*	1.14−3.02		

Separate analyses for women and men.

Model 1: Crude analysis for each variable.

Model 2: This model includes the variables responsibility for domestic work, relative socioeconomic position and psychological distress at age 21.

† No result presented because of few cases.

The ORs for psychological distress in relation to the combined variable responsibility for domestic work and perception of gender equality in the couple relationship are presented in Table 3. In relation to the reference group, we found statistically significantly higher ORs for “less than half and not gender-equal” in the bivariate and multivariate analysis. There were also higher ORs for the group “more than half and not gender-equal” in the bivariate analysis and when gender was adjusted for. After additional adjustments for earlier psychological distress, the association turned statistically borderline significant.

The ORs for psychological distress in relation to the combined variable responsibility for domestic work and relative socioeconomic position are presented in Table 4. In relation to the reference group, there were statistically significantly higher ORs for the group “less than half and partner higher socioeconomic position” in the bivariate and multivariate analysis. There were also higher ORs for “more than half and equal socioeconomic position” in the bivariate analysis and when gender was adjusted for. After additional adjustments for earlier psychological distress, the association turned statistically borderline significant.

**Table 3:** Psychological distress (age 42) in relation to the combined variable responsibility for domestic work and perception of gender equality in the couple relationship.

	Model 1		Model 2		Model 3		Model 4		n	nwomen/men	%women/men
	OR	95% CI	OR	95% CI	OR	95% CI	OR	95% CI			
Half of the responsibility *–* gender-equal (reference)	1.00	-	1.00	-	1.00	-	1.00	-	149	61/88	41/59
Half of the responsibility *–* not gender-equal	1.47	0.84−2.58	1.57	0.88−2.77	1.49	0.83−2.68	1.48	0.82−2.65	112	37/75	33/67
Less than half *–* gender-equal	1.76	0.81−3.80	2.11	0.96−4.64	2.12	0.95−4.72	2.20	0.99−4.92	39	7/32	18/82
Less than half – not gender-equal	2.16*	1.22−3.82	2.89*	1.57−5.34	3.07*	1.64−5.74	3.05*	1.63−5.70	92	3/89	3/97
More than half *–* gender-equal	0.69	0.33−1.44	0.57	0.27−1.20	0.49	0.23−1.06	0.48	0.22−1.05	73	50/23	68/32
More than half – not gender-equal	2.39*	1.48−3.86	1.72*	1.02−2.76	1.69	0.99−2.89	1.69	0.99−2.89	200	185/15	93/7

Logistic regression analysis (ORs and 95% CIs).

Model 1: Crude analysis.

Model 2: Adjusted for gender.

Model 3: Adjusted for gender and psychological distress at age 21.

Model 4: Adjusted for gender, psychological distress at age 21 and number of children.

* p≤0.05.

**Table 4:** Psychological distress (age 42) in relation to the combined variables responsibility for domestic work and relative socioeconomic position.

	Model 1		Model 2		Model 3		Model 4		n	nwomen/men	%women/men
	OR	95% CI	OR	95% CI	OR	95% CI	OR	95% CI			
Half of the responsibility – equal sei (reference)	1.00	-	1.00	-	1.00	-	1.00	-	155	56/99	56/99
Half of the responsibility – individual higher SEI	0.72	0.32−1.62	0.73	0.32−1.66	0.70	0.30−1.62	0.72	0.31−1.66	49	16/33	33/67
Half of the responsibility – partner higher SEI	1.76	0.92−3.37	1.64	0.85−3.18	1.68	0.86−3.29	1.66	0.85−3.25	59	28/31	47/52
Less than half – equal SEI	1.70	0.93−3.09	2.14*	1.14−4.00	2.23*	1.18−4.24	2.26*	1.19−4.29	75	6/69	8/92
Less than half – individual higher SEI	1.06	0.39−2.88	1.33	0.48−3.66	1.38	0.49−3.89	1.34	0.48−3.76	24	2/22	8/92
Less than half – partner higher SEI	3.19*	1.43−7.14	4.09*	1.79−9.35	4.63*	1.99−10.76	4.87*	2.09−11.35	30	2/28	7/93
More than half – equal SEI	1.85*	1.12−3.05	1.33*	1.78−2.28	1.28	0.74−2.23	1.28	0.73−2.23	147	124/23	85/15
More than half – individual higher SEI	1.60	0.84−3.00	1.07	0.58−2.11	1.10	0.36−1.61	1.10	0.55−2.20	66	62/4	94/6
More than half – partner higher SEI	1.11	0.56−2.23	0.81	0.39−1.67	0.76	0.30−0.75	0.75	0.36−1.60	58	47/11	81/19

Logistic regression analysis (ORs and 95% CIs).

Model 1: Crude analysis.

Model 2: Adjusted for gender.

Model 3: Adjusted for gender and psychological distress at age 21.

Model 4: Adjusted for gender, psychological distress at age 21 and number of children.

* p≤0.05.

## Discussion

This study showed that the relation between responsibility for domestic work and psychological distress was influenced by gender inequality in the couple relationship and relative socioeconomic position. Higher ORs for psychological distress were found for the combination of ‘unequal responsibility for domestic work and experiencing the relationship as gender-unequal’, as well as for ‘having less than half of the responsibility for domestic work and partner higher socioeconomic position’.

As documented in earlier studies, inequality in the division of domestic work is associated with higher risks of psychological distress [8]–[10]. However, the results of this study indicate that it is not only a matter of whether the responsibility for domestic work is equal or not, but also the relational context in which the responsibilities are divided within the couple relationship. Unequal responsibility for domestic work in combination with a perception of gender equality in the couple relationship was not associated with psychological distress, while the combination of unequal responsibility and gender inequality in the couple relationship was. These findings can be understood in relation to the experiences of domestic work as equal or fair. If the division of domestic work is not in accordance with our expectations, e.g. if we experience the division as unequal when we expected it to be equal, there is a risk of feeling deprived which thereby increases the risk of psychological ill health [16], [23]. However, in earlier research it is well documented that an unequal division of domestic work can be regarded as fair [16], [23], [24]. One reason for this could be a subordinate position; e.g. being in economic dependency on a partner can contribute to a greater acceptance of inequalities [23]. Research has shown that women and men avoid describing an unequal division of domestic work in terms of gender inequality [16], [17], [25]. Heterosexual couples often internalize a view of gender equality where the women do the majority of the domestic work. To avoid viewing one’s relationship as gender-unequal could be a strategy to evade conflicts and psychological ill health in the short run [23]. However, inequality can take two different directions, which in our study was expressed through women having a greater responsibility and men a smaller responsibility for domestic work. As shown in this paper, both these directions of inequality in domestic work, in combination with experiencing the couple relationship as gender-unequal, were associated with psychological distress, something that notably exacerbates the existing gender differences in well-being [26].

Earlier research on domestic work has found a socioeconomic gradient in psychological distress. Among women taking more than half of the responsibility for domestic work, those in lower socioeconomic positions have been shown to have higher risks of psychological distress compared to women in higher socioeconomic positions [5]. In this study, women were in the majority (85%) in the combination of having more than half of the responsibility for domestic work and an equal socioeconomic position to the partner, which was associated with higher ORs of psychological distress. Even though adjustments were made for gender, the overrepresentation of women indicates that these results are based on a situation where mainly women are found. To understand these results it is important to recognize that domestic work is part of the gendered division of labour, which implies that domestic work is mainly performed by women while women’s paid work is segregated and financially lower valued than the paid work of men [6], [18]. Within heterosexual couples, a situation where women have greater responsibility for domestic work and an equal socioeconomic position to their partner expresses partially asymmetrical gendered power relations, reproducing traditional arrangements of domestic work [27]. However, even if the socioeconomic positions of the couples are equal, the Swedish labour market is to a high degree gender-segregated, with lower salaries and worse work environment conditions for many women [6]. Having the same socioeconomic position as the partner might therefore not imply the same income, working conditions or power in the labour market. However, the associations between relative socioeconomic position and psychological distress were generally stronger among men than among women, which might be related to the fact that measurements of socioeconomic position tend to capture inequalities among men more precisely than among women [11], [28]. Furthermore, both having less than half of the responsibility for domestic work and lower socioeconomic position than the partner was independently associated with higher ORs of psychological distress. It is therefore reasonable that the combination of these two variables also was associated with psychological distress. As this combined group consisted almost entirely of men (93%), we interpret these findings as mainly representative for men. One explanation for these results could be that men with a lower socioeconomic position than the partner might challenge the gender order, which per se can be damaging to health in the short run [29].

As it is suggested to use not only occupation as an individual measure of socioeconomic position [12], we used a relational approach to capture the power relation within the couples. However, socioeconomic position has been shown to be measured with less precision among women than among men and can therefore have different meanings for women and men [11], [28]. One explanation for this could be that women’s and men’s position in the Swedish labour market are very distinct in the distribution of occupational classifications, and they have different access to social, psychological and material resources [6], [11]. The different meaning of socioeconomic positions for women and men may be related to our finding that men with a lower socioeconomic position than their partner have higher risks of psychological distress, whereas there was no such relation among women. Furthermore, our results on the combination of domestic work and relative socioeconomic position stress the importance of regarding socioeconomic position as relational, and that gender might not be the only power relation to consider in analyses of domestic work and psychological distress. By analysing how domestic work interacts with perceptions of gender equality in the couple relationship and relative socioeconomic position, new patterns of the relation between domestic work and psychological distress can become visible, and thereby contribute to increasing our knowledge of social inequalities in health.

### On the Method

As many of the earlier studies have a cross-sectional design [2], [30], it is difficult to ascertain whether gender equality precedes good health or if good health is a prerequisite for gender equality. The strengths of this study are the high quality of the data, with a very high response rate and the longitudinal design which enabled adjustments for earlier health status. The results of this study are based on data from the Northern Swedish Cohort, which has been shown to be representative of Swedish society with respect to sociodemographics, health behaviours and health status [19]. However, the Swedish context is characterized by a high participation rate of women in the paid workforce and public support for gender equality in all spheres of life [31], conditions that may be important for the results found in this study. A limitation is that this study is based on a small sample, which increased the risks of false negative results (type 2 error) in the statistical analysis and made it impossible to do a more detailed gender analysis, i.e. separate analyses for women and men. Future research is required to conduct the analysis on a larger sample from different countries and with separate analyses for women and men.

### Conclusions

This study highlights the importance of the relation to a partner, i.e. the perception of gender equality in the couple relations and relative socioeconomic position, for the association between domestic work and psychological distress. This study showed that domestic work is a highly gendered activity as women tend to have a greater and men a smaller responsibility. Both these directions of inequality in domestic work, in combination with experiencing the couple relationship as gender-unequal, were associated with psychological distress. There is a need for more research with a relational approach on inequalities in health in order to capture the power relations within couples in various settings.

## References

[1] Lahelma E, Arber S, Kivela K, Roos E (2002). Multiple roles and health among British and Finnish women: the influence of socioeconomic circumstances.. Soc Sci Med.

[2] Artazcoz L, Borrell C, Cortes I, Escribà-Agüir V, Cascant L (2007). Occupational epidemiology and work related inequalities in health: a gender perspective for two complementary approaches to work and health research.. J Epidemiol Community Health.

[3] Evertsson LN, Nyman C (2009). If not negotiation, then what? Gender equality and the organization of everyday life in Swedish couples.. Interpersona.

[4] Staland Nyman C, Alexanderson K, Hensing G (2008). Associations between strain in domestic work and self-rated health: a study of employed women in Sweden.. Scand J Public Health.

[5] Matthews S, Power C (2002). Socio-economic gradients in psychological distress: a focus on women, social roles and work-home characteristics.. Soc Sci Med.

[6] Statistics Sweden (2010). Women and men in Sweden.. http://www.scb.se/Pages/PublishingCalendarViewInfo____259923.

[7] Evertsson M, England P, Mooi-Reci I, Hermsen J, Bruijn J (2009). Is Gender Inequality Greater at Lower or Higher Educational Levels? Common Patterns in the Netherlands, Sweden, and the United States.. Social Politics.

[8] Bird CE (1999). Gender, household labor, and psychological distress: The impact of the amount and division of housework.. J Health Soc Behav.

[9] Väänänen A, Kevin MV, Ala-Mursula L, Pentti J, Kivimäki M (2004). The double burden of and negative spillover between paid and domestic work: associations with health among men and women.. Women Health.

[10] Harryson L, Novo M, Hammarström A (2012). Is gender inequality in the domestic sphere associated with psychological distress among women and men? Results from the Northern Swedish Cohort.. J Epidemiol Community Health.

[11] MacIntyre S, Hunt K (1997). Socio-economic Position, Gender and Health.. Journal of Health Psychology.

[12] Annandale E, Hunt K, Annandale E, Hunt K (2001). Gender Inequalities in Health: Research at the crossroads.. Buckingham: Open University Press.

[13] Evertsson M, Nermo M (2007). Changing Resources and the Division of Housework: A Longitudinal Study of Swedish Couples.. European Sociological Review.

[14] Bartley M, Martikainen P, Shipley M, Marmot M (2004). Gender differences in the relationship of partner's social class to behavioural risk factors and social support in the Whitehall II study.. Soc Sci Med.

[15] Claffey ST, Mickelson KD (2009). Division of Household Labor and Distress: The Role of Perceived Fairness for Employed Mothers.. Sex Roles.

[16] Nordenmark M, Nyman C (2003). Fair or unfair? Perceived fairness of household division of labour and gender equality among women and men - The Swedish case.. European Journal of Women's Studies.

[17] Magnusson E, Magnusson E, Rönnblom M, Silius H (2008). Conflict, danger and difference. Nordic heterosexual couples converse about gender equality and fairness..

[18] Connell R (2009). *Gender. In world perspective.*. Cambridge: Polity Press.

[19] Hammarström A, Janlert U (2011). Cohort Profile: The Northern Swedish Cohort.. International Journal of Epidemiology: Accepted.

[20] Statistics Sweden (1983). Socioekonomisk indelning (SEI). Meddelande i samordningsfrågor 1982:4 [Swedish socioeconomic classifications].. Stockholm: Statistics Sweden.

[21] Reine I, Novo M, Hammarström A (2008). Does transition from an unstable labour market position to permanent employment protect mental health? Results from a 14-year follow-up of school-leavers.. BMC Public Health.

[22] Statistics Sweden (1980). Swedish survey of living conditions.. Stockholm: Statistics Sweden.

[23] Lennon MC, Rosenfield S (1994). Relative Fairness and the Division of Housework: The Importance of Options.. American Journal of Sociology.

[24] Braun M, Lewin-Epstein N, Stier H, Baumgärtner MK (2008). Perceived Equity in the Gendered Division of Household Labor.. Journal of Marriage and Family.

[25] Ahlberg J, Roman C, Duncan S (2008). Actualizing the "Democratic family"? Swedish policy rhetoric versus family practices.. Social Politics.

[26] Boye K (2008). Time spent on working. Is there a link between time spent on paid work and housework and the gender differences in psychological distress? In Boye K Happy hour? Studies on well being and time spent on paid and unpaid work.. Stockholm University: Dissertation.

[27] Magnusson E (2005). Gendering or Equality in the Lives of Nordic Heterosexual Couples with Children: No Well Paved Avenues Yet.. Nordic Journal of Feminist and Gender Research.

[28] Mustard CA, Etches J (2003). Gender differences in socioeconomic inequality in mortality.. J Epidemiol Community Health.

[29] Backhans MC, Burstrom B, Lindholm L, Månsdotter A (2009). Pioneers and laggards - Is the effect of gender equality on health dependent on context?. Soc Sci Med.

[30] Månsdotter A, Lindholm L, Lundberg M, Winkvist A, Öhman A (2006). Parental share in public and domestic spheres: a population study on gender equality, death, and sickness.. J Epidemiol Community Health.

[31] SOU (2005). Makten att forma samhället och sitt eget liv - jämställdhetspolitiken mot nya mål 06/155 [Official Report of the Swedish Government: Power to shape society and your own life - towards new gender policy objectives].. http://www.regeringen.se/sb/d/108/a/47912.

